# Acute Flaccid Paralysis Followed by Guillain-Barré Syndrome in West Nile Virus Infection: A Case Report

**DOI:** 10.7759/cureus.114438

**Published:** 2026-08-12

**Authors:** Yae Eun Kim, Michael J Suh

**Affiliations:** 1 College of Osteopathic Medicine, New York Institute of Technology, New York, USA; 2 Neurology, American Board of Psychiatry and Neurology, New York, USA; 3 Neurology, American Academy of Neurology, New York, USA; 4 Neurology, American Society of Electrophysiology, New York, USA; 5 Neurology, Child Neurology Society, New York, USA; 6 Neurology, American Academy of Sleep Medicine, New York, USA

**Keywords:** acute flaccid paralysis, case report, differential diagnosis, guillain-barré-like syndrome, guillain-barre syndrome (gbs), mri images, neuroinvasive west nile virus, west nile-associated acute flaccid paralysis, west nile virus, west nile virus infection

## Abstract

Neuroinvasive West Nile virus infection can present with a broad spectrum of neurologic manifestations, creating diagnostic and management challenges. We report a case that initially presented with acute flaccid paralysis and subsequently evolved to include features consistent with Guillain-Barré syndrome during hospitalization. This case highlights the dynamic nature of neuromuscular involvement in neuroinvasive West Nile virus infection and the importance of ongoing clinical reassessment as neurologic findings evolve.

## Introduction

West Nile virus is an arbovirus, initially isolated from the West Nile District of Uganda, Africa, in 1937, that has expanded its geographical range globally [1,2]. Transmitted through a mosquito vector, it was first detected in the United States in 1999 in Queens, New York City, and since then it has established itself as one of the most widespread arboviruses worldwide, with over 48,000 reported cases in the U.S. alone and persistent seasonal outbreaks [1-4]. Despite its global burden, about 80% of infected patients are asymptomatic and about 20% present with a self-limited febrile illness [3,4]. Only about 1-2% of infections in immunocompetent people progress to neuroinvasive disease, and even fewer develop atypical peripheral nervous system manifestations [3-7]. Up to 75% of those with WNV neuroinvasive disease suffer from long-term physical, cognitive, and functional sequelae [4,5]. Though the true incidence remains uncertain, studies show advanced age being the most significant risk factor among many others, including cardiovascular disease, chronic kidney disease, malignancies, and immunocompromised states [4]. We present a case of seropositive WNV neuroinvasive infection that initially presented with acute flaccid myelitis, with later development of clinical and electrodiagnostic features consistent with Guillain-Barré syndrome over 10 months of continued care, resulting in long-term neurologic sequelae.

## Case presentation

A 73-year-old man with a history of chronic hypertension, diabetes mellitus, prior cardiac arrest, and coronary artery bypass grafting in 2024 presented to an outpatient neurology clinic in a wheelchair for persistent lower extremity weakness and intermittent tremors following West Nile virus infection in March 2026. Upon receiving past medical history, it was evident that the patient presented with atypical progression of neuroinvasive West Nile virus infection that might be contributing to his signs and symptoms.

The patient first presented to an emergency department in Switzerland in late August of 2025 with lower extremity weakness and numbness following several days of worsening fatigue, chills, and generalized myalgia. Primary flu-like symptoms reportedly began a few days prior to traveling from Queens, New York, to Switzerland. His home medications included aspirin 81 mg, metoprolol succinate 25 mg, and atorvastatin 80 mg daily.

On initial presentation to a Swiss emergency room several days after flu-like symptoms. Examination revealed diffuse cutaneous and intraoral petechiae with resting tremor that worsened to targeted tremor. Labs initiated in the Swiss emergency room were notable for thrombocytopenia. Hospital admission was recommended, but the patient left against medical advice. The following day, he returned to the emergency department with worsening lower extremity weakness and numbness with severe vertigo and gait difficulty. He denied a gradual ascending pattern of weakness. He was transferred to a larger local hospital for evaluation.

**Figure 1:**
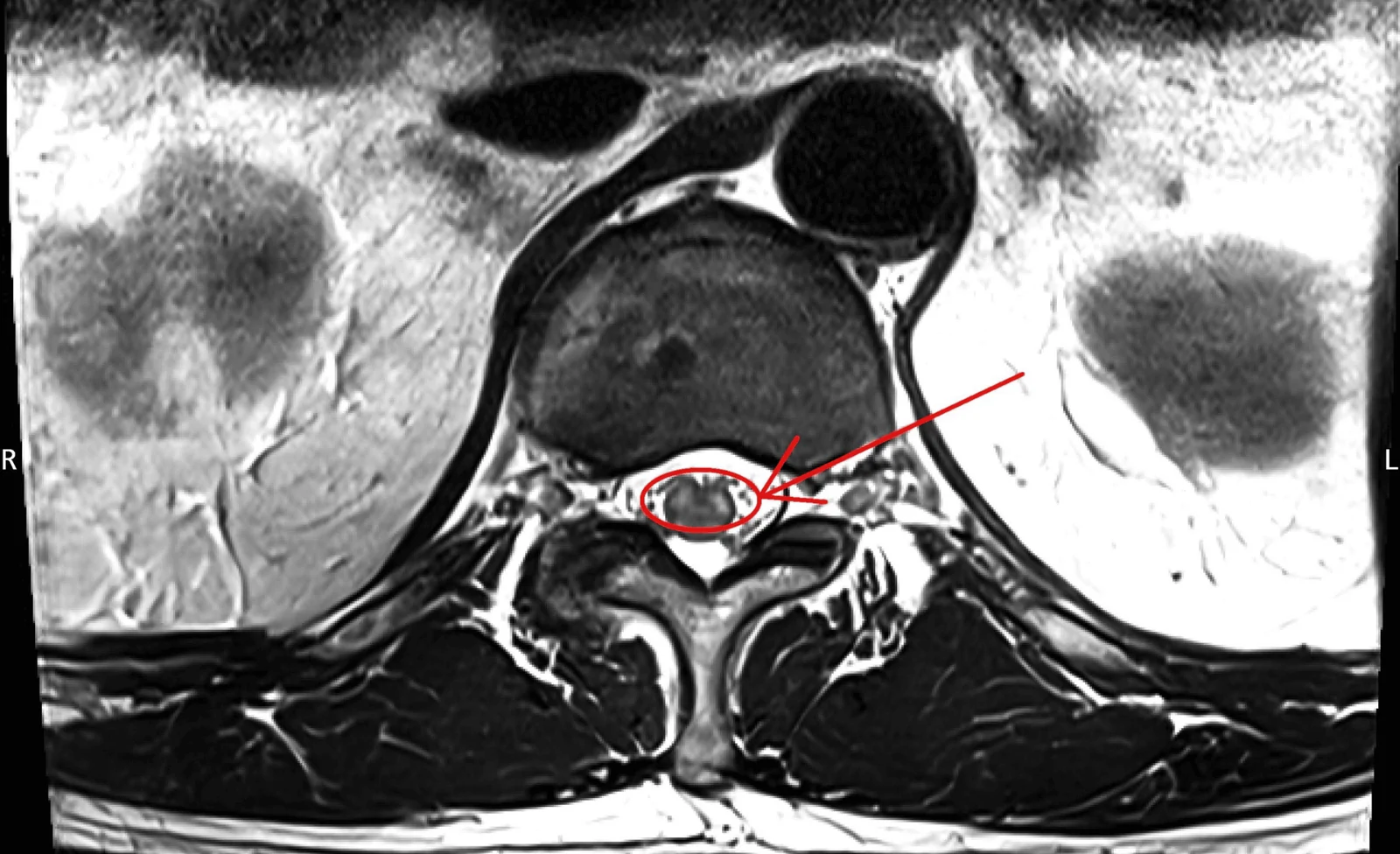
Axial T2-weighted MRI of the thoracic/lumbar spine Axial T2-weighted MRI image at the T12/L1 level demonstrates a focal intramedullary hyperintense signal abnormality involving the anterior gray matter/anterior horn region, indicated by an arrow. T2-weighted images specifically highlight water and fluids to find abnormalities like swelling or inflammation in comparison to an MRI. This finding is supportive of anterior horn cell involvement in the setting of suspected West Nile virus–associated acute flaccid myelitis.

During hospitalization in the local Swiss hospital, he remained alert and oriented to person, place, and time and did not require ICU admission or intubation throughout his stay. Initial diagnostic workup was notable for viral or post-viral meningitis (Table 1). Brain MRI demonstrated periventricular accentuated fluid-attenuated inversion recovery (FLAIR)-hyperintense white matter lesions in corona radiata, consistent with chronic microangiopathy. Spinal MRI was ordered and performed in the Swiss hospital, and imaging showed T2-hyperintense signal abnormalities involving anterior horns from spinal level T11/T12 to conus, consistent with acute flaccid myelitis or anterior horn cell disease (Figure 1).

**Table 1:** Cerebrospinal fluid findings from lumbar puncture in Switzerland Cerebrospinal fluid analysis demonstrated pleocytosis with elevated protein, lactate, albumin, and immunoglobulin levels, while glucose remained within the laboratory reference range. These findings were consistent with inflammatory central nervous system involvement in the setting of suspected viral or post-viral meningitis.

Lumbar Puncture – CSF Findings		
Test	Result	Reference Range
Total cell count	118 cells/µL	≤5 cells/µL
Erythrocytes	<1 cells/µL	NA
Mononuclear cells	55.1%; absolute count 65 cells/µL	NA
Polymorphonuclear cells	44.9%; absolute count 53 cells/µL	NA
Protein	1.71 g/L	0.20–0.40 g/L
Glucose	2.66 mmol/L	2.22–3.89 mmol/L
Lactate	4.47 mmol/L	1.00–2.10 mmol/L
Albumin	1,200 mg/L	110–350 mg/L
IgG	243.0 mg/L	10.0–40.0 mg/L
IgA	77.40 mg/L	0.50–6.00 mg/L
IgM	33.40 mg/L	0.05–0.80 mg/L

He was admitted for further treatment and management of viral meningitis. Empiric treatment with ceftriaxone and doxycycline for tick-borne illness was initiated, but tick-borne viral etiologies were later ruled out after negative lab results (Table 2). He received broad-spectrum antibiotics and antivirals - amoxicillin and acyclovir - and supportive care with pregabalin. The patient’s lower extremity numbness improved during hospitalization; however, lower extremity weakness persisted.

**Table 2:** Laboratory and cerebrospinal fluid findings in Switzerland The key laboratory and cerebrospinal fluid (CSF) findings support the diagnosis of West Nile neuroinvasive disease. CSF analysis demonstrated pleocytosis with a mixed mononuclear and polymorphonuclear predominance, elevated protein and lactate levels, and evidence of blood-CSF barrier dysfunction. Initial West Nile virus (WNV) serology showed positive IgM with negative IgG, followed by subsequent IgG seroconversion with persistent IgM positivity. An extensive infectious workup, including CSF testing for common bacterial and viral pathogens, was otherwise negative.

Laboratory parameter	Result	Reference range/interpretation
Peripheral blood		
Leukocyte count (27 Aug 2025)	11.66 × 10⁹/L	3.00–10.50 × 10⁹/L
Leukocyte count (28 Aug 2025)	14.74 × 10⁹/L	3.00–10.50 × 10⁹/L
Absolute neutrophil count (28 Aug 2025)	11.88 × 10⁹/L	1.60–7.40 × 10⁹/L
C-reactive protein (27 Aug 2025)	4 mg/L	<5 mg/L
C-reactive protein (28 Aug 2025)	29 mg/L	<5 mg/L
Cerebrospinal fluid (27 Aug 2025)		
Appearance	Colorless, clear	—
Leukocyte count	118 cells/µL	≤5 cells/µL
Erythrocyte count	<1 cell/µL	—
Mononuclear cells	55.1% (65 cells/µL)	—
Polymorphonuclear cells	44.9% (53 cells/µL)	—
Protein	1.71 g/L	0.20–0.40 g/L
Glucose	2.66 mmol/L	2.22–3.89 mmol/L
Lactate	4.47 mmol/L	1.00–2.10 mmol/L
Albumin	1,200 mg/L	110–350 mg/L
CSF/serum albumin quotient	26.91	<9.00
Oligoclonal bands	Type 4 (identical bands in CSF and serum)	—
West Nile virus serology		
WNV IgG (28 Aug 2025)	Negative (index 0.1)	<0.8
WNV IgM (28 Aug 2025)	Positive (index 5.1)	<0.8
WNV IgG (12 Sep 2025)	Positive (index 1.3)	<0.8
WNV IgM (12 Sep 2025)	Positive (index 2.8)	<0.8
Selected infectious workup		
CSF meningitis/encephalitis PCR panel	Negative	No pathogen detected
Blood cultures	Negative	No growth
Borrelia burgdorferi IgG/IgM	Negative	—
Tick-borne encephalitis virus IgG/IgM	Negative	—
HSV-1/HSV-2 PCR	Negative	—
VZV PCR	Negative	—
Enterovirus PCR	Negative	—
Cytomegalovirus PCR	Negative	—
Human herpesvirus 6 PCR	Negative	—
HIV-1/2 screening	Negative	—
Treponema pallidum screening	Negative	—

Once clinically stable, the patient was cleared for a direct transfer back to New York in mid-September 2025. Immediately upon arrival, he was directly admitted to a hospital in Manhattan, New York, presenting with persistent bilateral lower extremity weakness, with significant worsening in the left lower extremity compared to what he felt before the direct transfer from Switzerland. Upon presentation, vital signs were stable. Neurologic physical exam revealed right gaze nystagmus, anisocoria, and decreased strength in bilateral lower extremities with relative preservation of distal right lower extremity strength at 5/5 (Table 3).

**Table 3:** Comprehensive neurological examination findings at admission in NYC (~21 days post-flu-like illness) Neurological examination demonstrated preserved upper extremity strength with marked bilateral lower extremity weakness, more pronounced proximally. Sensory modalities, including light touch, temperature, and vibration, were intact in all four extremities. Deep tendon reflexes were preserved in the upper extremities but absent at the patellar and Achilles tendons bilaterally, with mute plantar responses. Motor strength was graded on the Medical Research Council (MRC) scale (0/5 to 5/5). Deep tendon reflexes graded on a standard 0 to 4+ scale. Sensory findings were evaluated as light touch and pinprick modalities across major dermatomes.

Examination Component and Modality	Left Side Finding	Right Side Finding
MOTOR STRENGTH (0-5 SCALE)		
Deltoid	5/5	5/5
Biceps	5/5	5/5
Triceps	5/5	5/5
Hand Grip	5/5	5/5
Hip Flexion	1/5	1/5
Hip Extension	2/5	2/5
Knee Flexion	2/5	3/5
Knee Extension	2/5	3/5
Ankle Dorsiflexion	2/5	3/5
Ankle Plantarflexion	2/5	3/5
Plantar Response	Mute	Mute
DEEP TENDON REFLEXES (0-4 SCALE)		
Biceps	2+	2+
Triceps	2+	2+
Brachioradialis	2+	2+
Patellar	0	0
Achilles (Ankle Jerk)	0	0
SENSORY EXAMINATION		
Light Touch	Intact in all four extremities	Intact in all four extremities
Temperature	Intact in all four extremities	Intact in all four extremities
Vibration	Intact in all four extremities	Intact in all four extremities

Upon admission to the hospital post return to NYC, mental status examination was unremarkable; he was alert and oriented to person, place, and time, with intact decision-making capacity. Subsequent neurological physical exam demonstrated diminished deep tendon reflexes and gross motor strength in bilateral lower extremities, worse on the left. Sensation to light touch, vibration, and temperature remained intact. Gait examination could not be completed because the patient was unable to stand.

Initial laboratory evaluation at NYC was notable for mild anemia (HGB 13.3, hematocrit (CRIT) 38.7%), coagulation abnormalities (prothrombin time (PT) 18.2, international normalized ratio (INR) 1.6, activated partial thromboplastin time (APTT) 39.9), monocytosis consistent with chronic or viral inflammation (CBC differential: monocytes 12.5%), and hyponatremia (133-134). The patient was admitted to the inpatient neurology unit for further evaluation of tick paralysis, Guillain-Barré, and transverse myelitis

Inflammatory workup was remarkable for autoimmune disorder (positive ANA with speckled pattern at titer 1:160). Infectious workup was notable for West Nile virus IgG serology and QuantiFERON-TB Gold tuberculosis testing (Table 4). This was relatively consistent with the infectious workup done in Switzerland (Table 2). Blood cultures, Lyme panel, and CT chest, abdomen, and pelvis obtained in the setting of a positive QuantiFERON-TB Gold test were unremarkable.

**Table 4:** Infectious disease and cerebrospinal fluid (CSF) diagnostic panel Serologic testing was notable for positive West Nile virus IgG and positive QuantiFERON-TB Gold testing, while Lyme serology and cerebrospinal fluid meningitis/encephalitis PCR panel were negative for common bacterial, viral, and fungal pathogens.

Diagnostic Test Name	Patient Result	Reference Range/Expected Result	Clinical Interpretation
West Nile virus antibody IgG	3.21	Negative	Positive
West Nile virus antibody IgM	5.23	Negative	Positive
QuantiFERON-TB Gold	Positive	Negative	Positive
Borrelia burgdorferi antibody ELISA	Negative	Negative	Negative
Escherichia coli K1, CSF PCR	Negative	Negative / Not detected	Not detected
Haemophilus influenzae, CSF PCR	Negative	Negative / Not detected	Not detected
Listeria monocytogenes, CSF PCR	Negative	Negative / Not detected	Not detected
Neisseria meningitidis, CSF PCR	Negative	Negative / Not detected	Not detected
Group B Streptococcus, CSF PCR	Negative	Negative / Not detected	Not detected
Streptococcus pneumoniae, CSF PCR	Negative	Negative / Not detected	Not detected
Cytomegalovirus, CSF PCR	Negative	Negative / Not detected	Not detected
Herpes simplex virus 1, CSF PCR	Negative	Negative / Not detected	Not detected
Herpes simplex virus 2, CSF PCR	Negative	Negative / Not detected	Not detected
Enterovirus, CSF PCR	Negative	Negative / Not detected	Not detected
Human parechovirus, CSF PCR	Negative	Negative / Not detected	Not detected
Varicella-zoster virus, CSF PCR	Negative	Negative / Not detected	Not detected
Cryptococcus neoformans/gattii, CSF PCR	Negative	Negative / Not detected	Not detected

Lumbar puncture showed cerebrospinal fluid pleocytosis, elevated protein levels, and normal glucose (Table 5), consistent with viral meningitis or post-viral central nervous system inflammation. Additional testing was ordered for peripheral neuropathy. Electromyography demonstrated bilateral L5-S1 radiculopathy.

**Table 5:** Lumbar puncture cerebrospinal fluid findings Lumbar puncture demonstrated mild cerebrospinal fluid pleocytosis, elevated protein, and normal glucose, with a decreasing red blood cell count across tubes suggestive of possible traumatic tap as indicated by the arrows in the blood cell count results.

CSF parameter	Result	Unit	Reference range	Interpretation
White blood cell count	21 → 18	cells/µL	0–5	Mild pleocytosis
Red blood cell count	146 → 6	cells/µL	0	Decreased across tubes; possible traumatic tap
Protein	170	mg/dL	15–45	Elevated
Glucose	60	mg/dL	40–70	Within normal range

**Figure 2:**
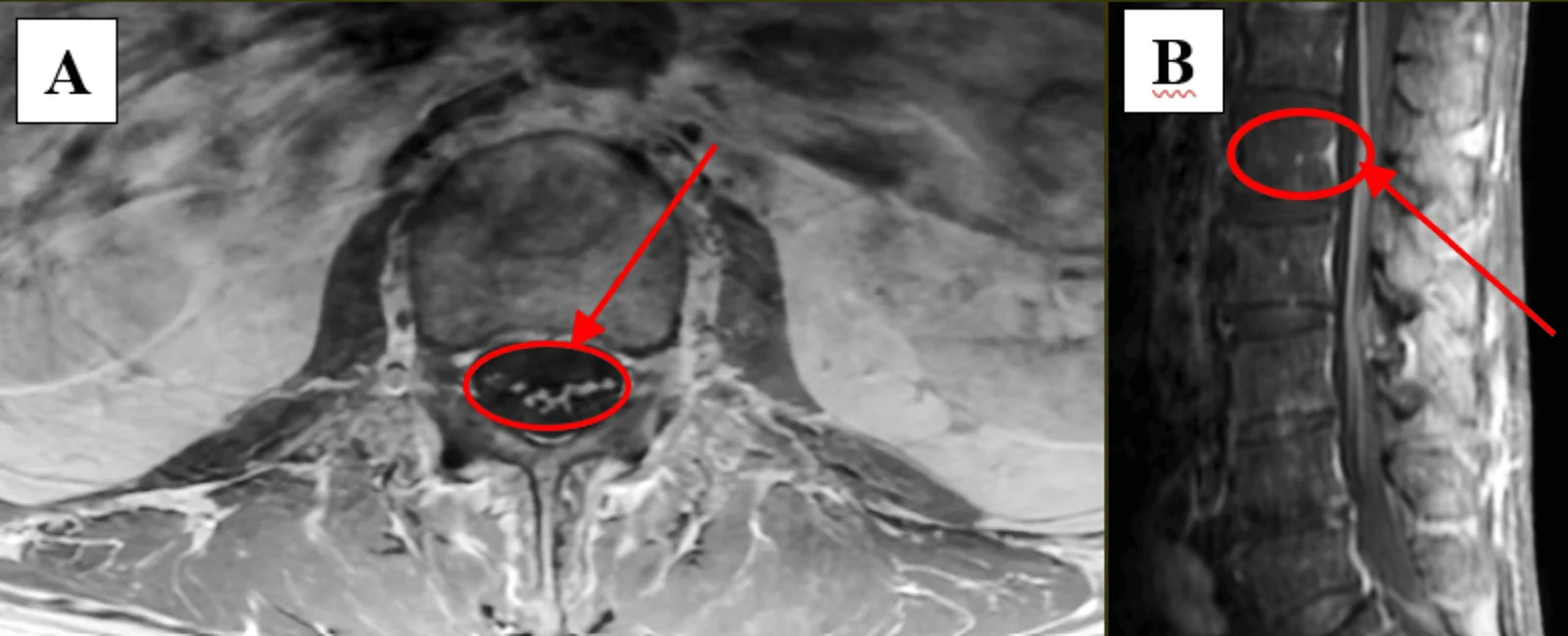
MRI findings suggestive of cauda equina nerve root involvement (A) Contrast-enhanced axial T1-weighted MRI of the lumbar spine demonstrates smooth enhancement of the cauda equina nerve roots, indicated by the arrow. (B) Sagittal lumbar spine MRI similarly demonstrates abnormal signal/enhancement along the cauda equina nerve roots. In the appropriate clinical context, these findings are supportive of inflammatory polyradiculoneuropathy and Guillain-Barré syndrome-like involvement.

Further neuroimaging studies in New York were notable for smooth enhancement of nerve roots from L1/L2 through the cauda equina, consistent with Guillain-Barré syndrome in the appropriate clinical context (Figure 2). Imaging also revealed multiple degenerative discogenic disease (C6-C7 severe bilateral spinal foraminal stenosis, L4-L5 mild bilateral spinal foraminal stenosis). Symmetric edema of bilateral paraspinal muscles was also noted, which was interpreted as indeterminate myositis versus denervation injury.

The patient was treated with a five-day course of intravenous immunoglobulin, with intensive physical therapy, occupational therapy, and acute inpatient rehabilitation to improve mobility in lower extremities. He reported some improvement in weakness following IVIG therapy; however, bilateral lower extremity strength remained diminished. He continued inpatient rehabilitation and therapy for the next four weeks and demonstrated sufficient improvement for discharge. At discharge, he required a rolling walker and wheelchair because he remained unable to ambulate without assistance. The patient was additionally prescribed acetaminophen 325 mg as needed for pain management.

In March 2026, the patient presented to an outpatient neurology clinic in a wheelchair for persistent lower extremity weakness and intermittent tremors following West Nile virus infection. On physical examination, blood pressure was elevated (130/75 mmHg). Neurologic examination revealed decreased muscle tone in bilateral lower extremities, absent patellar reflexes, and diminished plantar reflexes bilaterally. Given his cardiovascular history and persistent vertigo, carotid and transcranial Doppler ultrasound was ordered at the outpatient neurology clinic for cerebrovascular evaluation. Doppler studies showed internal carotid stenosis without significant hemodynamic changes, and the overall cerebrovascular workup was unremarkable. MRI brain without contrast was ordered and obtained to evaluate for post-infection chronic irreversible changes and possible etiologies for intermittent tremors. Imaging was noted with nonspecific scattered punctate microhemorrhages involving the right cerebellum, right thalamus, and right frontal matter, possibly related to chronic hypertension.

Routine electroencephalography was performed to rule out possible generalized seizure versus nonepileptic events following West-Nile infection. The EEG was unremarkable with no epileptiform activity identified. The patient was advised to follow up with cardiology for hypertension management and with primary care for diabetes management, as these comorbidities may have contributed to persistent vertigo. Given the extent of neurologic injury and limited recovery despite aggressive acute rehabilitation care, full recovery of independent ambulation was considered unlikely.

## Discussion

West Nile virus is maintained and distributed in an enzootic cycle, with humans serving as incidental hosts [1-4]. WNV initially replicates in keratinocytes, Langerhans cells, and dendritic cells following transmission through an infected mosquito vector [2,4,6]. In neuroinvasive symptomatic infection, studies support activation of pro-inflammatory pathways by infected immune cells, followed by viral dissemination into the bloodstream and eventual involvement of the central nervous system and visceral organs [4,6]. Further studies suggest that WNV relies on axonal transport to invade further into the central nervous system, causing spinal cord infection, neuronal injury, and acute flaccid paralysis [8]. Unfortunately, there are no medical measures that can completely isolate WNV zoonotic virus from humans.

**Figure 3:**
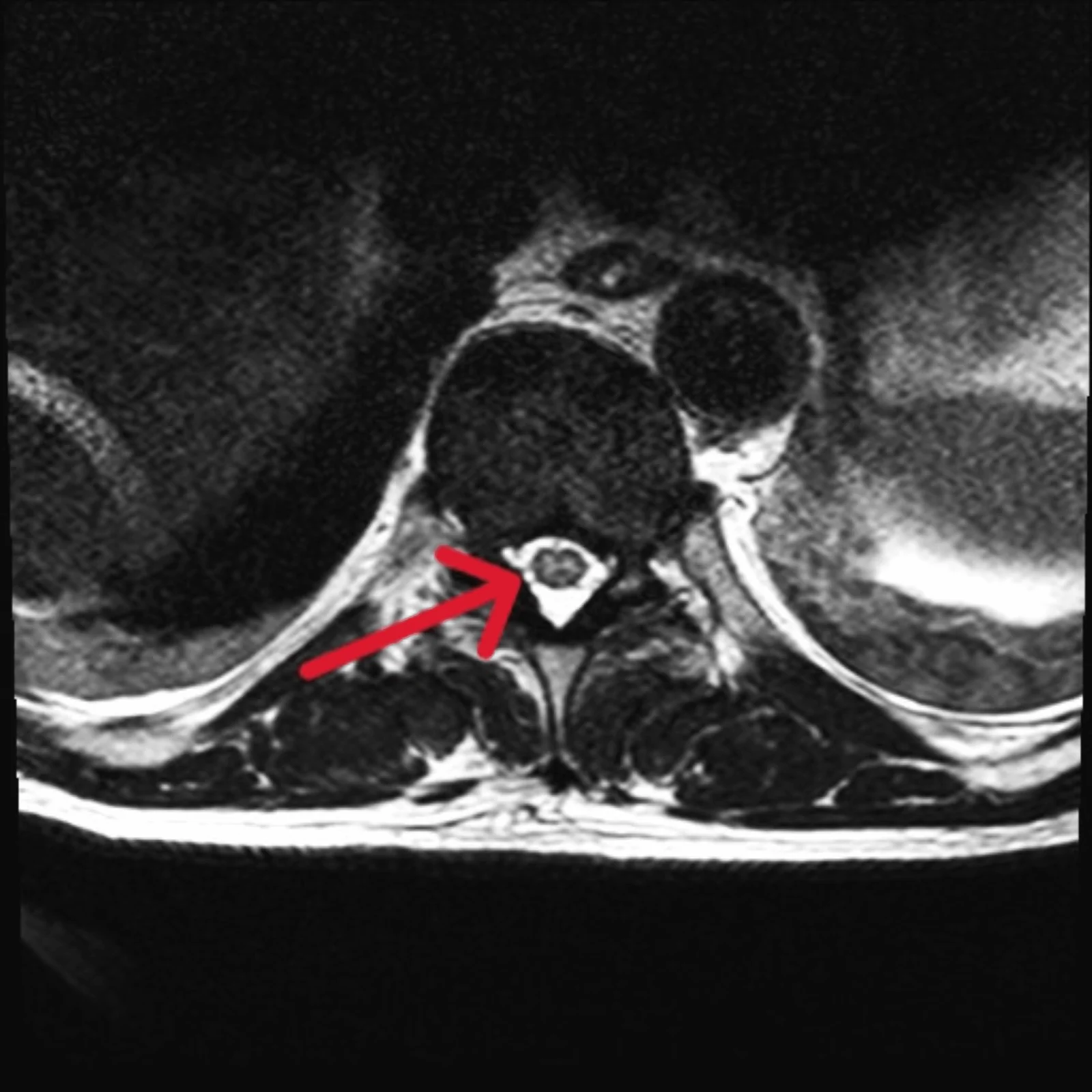
MRI findings of acute flaccid myelitis Spinal MRI demonstrates focal T2 hyperintensity involving the anterior gray matter/anterior horn region, supporting acute flaccid myelitis with anterior horn cell involvement. Adapted from Sapkota P [11]. West Nile virus encephalomyelitis. Radiopaedia.org case (rID: 174050), doi:10.53347/rID-174050, accessed June 1, 2026, used in accordance with the applicable Radiopaedia Creative Commons license.

Acute flaccid myelitis primarily affects the spinal cord, especially the anterior horn cells, leading to sudden muscle weakness and hyporeflexia [9,10]. Clinical presentation is notable for acute asymmetric lower motor neuron paralysis with relative preservation of sensation [9,10]. Its hallmark radiologic finding is focal T2 hyperintense lesions involving the anterior horns of the spinal cord (Figure 3) [9,10]. Its hypothesized mechanism involves viral-mediated inflammation and subsequent neuronal damage of motor neurons, resulting in impaired motor signal transmission and flaccid paralysis [8-10]. Because the injury preferentially involves the anterior horn cells, sensory pathways in the dorsal columns and posterior horns may be relatively spared, which can explain the presence of profound weakness with preserved sensation.

Transverse myelitis is an acute, acquired focal inflammatory disorder of the spinal cord characterized by rapid onset of motor weakness, sensory deficits, and autonomic dysfunction. Etiologies may be idiopathic, post-infectious, infectious, autoimmune, or associated with multifocal central nervous system demyelinating disease. The inflammatory process typically involves a segment of the spinal cord and predominantly produces bilateral neurologic deficits below the affected level, with the thoracic cord most commonly involved. Diagnostic findings often include T2-hyperintense spinal cord lesions on MRI, with or without gadolinium enhancement. Longitudinally extensive spinal cord lesions may be seen in certain inflammatory or demyelinating etiologies.

**Figure 4:**
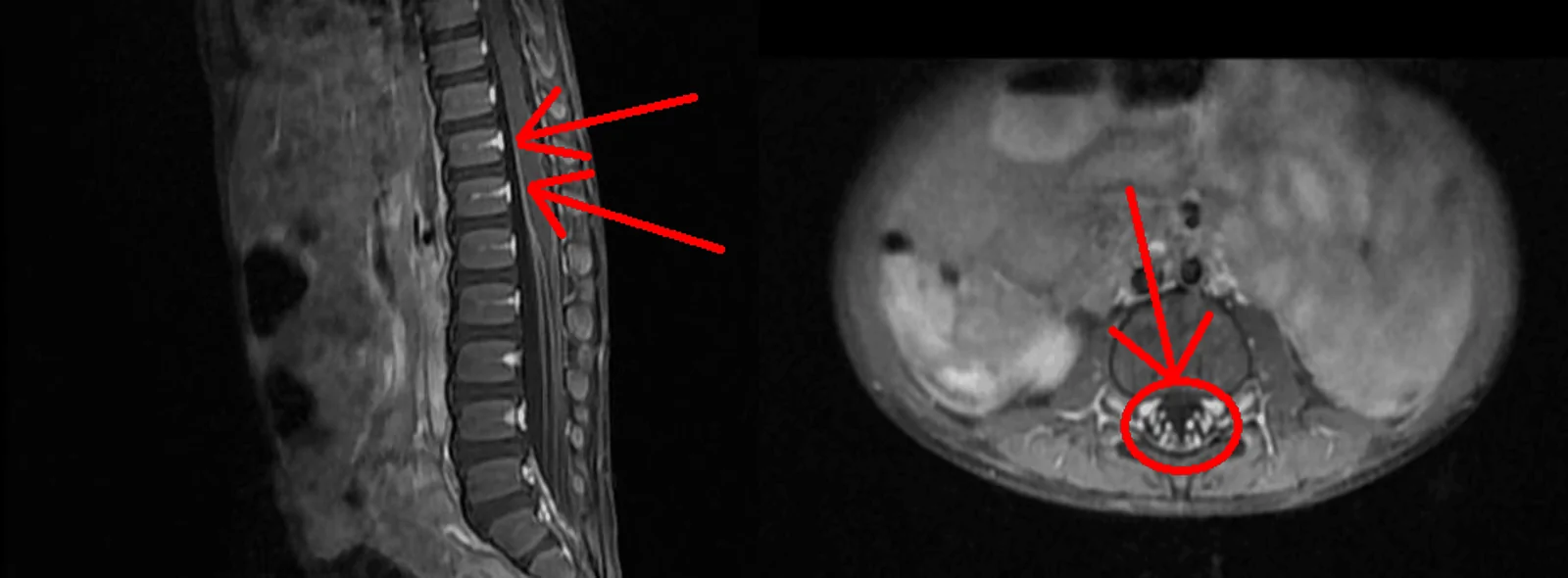
MRI findings of Guillain-Barré syndrome-like polyradiculoneuropathy Lumbar spine MRI demonstrates cauda equina nerve root enhancement/signal abnormality, supportive of inflammatory polyradiculoneuropathy in the appropriate clinical context. These findings highlight sequential central and peripheral nervous system involvement in neuroinvasive West Nile virus infection. Adapted from Zainab M [16]. Guillain-Barré syndrome. Radiopaedia.org case (rID: 211198), doi:10.53347/rID-211198, accessed June 1, 2026, with attribution in accordance with the applicable Radiopaedia Creative Commons license.

Guillain-Barré syndrome is an acute immune-mediated polyradiculopathy characterized by progressive weakness, diminished or absent reflexes, and albuminocytologic dissociation in the cerebrospinal fluid. It is commonly triggered by antecedent infection and may involve demyelination or axonal injury to the peripheral nervous system. Patients often present with numbness, distal paresthesias, limb pain, and progressive bilateral weakness that is typically relatively symmetric [13,14]. Supportive diagnostic findings may include electrodiagnostic evidence of polyradiculoneuropathy and contrast enhancement of the nerve roots, conus medullaris, or cauda equina on magnetic resonance imaging (Figure 4) [14,15].

Acute motor axonal neuropathy (AMAN), a variant of Guillain-Barré syndrome, is characterized by immune-mediated axonal injury predominantly affecting motor fibers while relatively sparing sensory fibers. AMAN contrasts with the more common demyelinating form of Guillain-Barré syndrome and is often associated with antecedent *Campylobacter jejuni* infection. Patients typically present with motor weakness and diminished or absent deep tendon reflexes, with minimal sensory deficits. Diagnosis is supported by cerebrospinal fluid findings of albuminocytologic dissociation and electrodiagnostic evidence of motor axonal involvement [13,17].

In this case, the patient is immunocompromised with diabetes mellitus type II and a positive QuantiFERON-TB Gold test. He also possesses additional risk factors: cardiovascular disease and history of immunosuppression post-coronary artery bypass grafting surgery. The patient initially developed clinical and radiographic findings consistent with WNV neuroinvasive disease manifesting as acute flaccid myelitis, with spinal imaging demonstrating anterior horn cell involvement. This pattern is consistent with the recognized tropism of WNV for motor neurons and its association with acute flaccid paralysis. However, approximately three weeks later, the patient developed findings suggestive of peripheral nerve root involvement, including diminished reflexes, persistent weakness, electrodiagnostic abnormalities, and smooth enhancement of the nerve roots extending from the lumbar spine to the cauda equina. These later findings were more consistent with a Guillain-Barré syndrome-like polyradiculoneuropathy.

Most reported neuromuscular manifestations of neuroinvasive WNV occur during the acute phase of infection and present as acute flaccid paralysis or a poliomyelitis-like syndrome [4,7]. Guillain-Barré syndrome-like presentations associated with WNV have been reported less frequently and are generally described as post-infectious or parainfectious peripheral nervous system complications [7,18]. The distinctive feature of this case is the apparent sequential pattern: an initial central nervous system process involving the anterior horn cells, followed by later evidence of peripheral nerve root involvement (Figure 3). The absence of persistent anterior horn signal abnormalities on later imaging, together with subsequent cauda equina nerve root enhancement, suggests a possible evolution from a spinal cord motor neuron syndrome to a peripheral polyradiculoneuropathy (Figure 4).

The pathophysiology underlying this possible evolution remains unclear. WNV neuroinvasion is thought to involve direct viral injury, inflammatory responses, gliosis, and neuronal loss within the central nervous system [4,6-8]. By contrast, Guillain-Barré syndrome is generally considered immune-mediated, often triggered by molecular mimicry or post-infectious immune activation [13,14]. This case raises the possibility that WNV may produce both direct neuroinvasive injury and delayed immune-mediated peripheral nervous system involvement in the same patient. Further study is needed to clarify whether such cases represent distinct sequential mechanisms, overlapping central and peripheral nervous system involvement, or an evolving post-infectious immune response.

This case also highlights the substantial long-term morbidity associated with WNV neuroinvasive disease [9,10]. Despite treatment with intravenous immunoglobulin and intensive rehabilitation, the patient continued to experience persistent lower extremity weakness, impaired ambulation, and functional limitations months after infection. These findings reinforce the importance of close neurologic follow-up, early rehabilitation, and careful monitoring for evolving neuromuscular complications in patients with WNV neuroinvasive disease.

Finally, this case emphasizes the need for heightened clinical vigilance in older patients and those with significant medical comorbidities who present with suspected WNV infection. Because treatment for West Nile virus meningitis remains primarily supportive, the practical implication of this case is not targeted antiviral therapy but early recognition of evolving neuromuscular complications. Serial neurologic examinations, timely electrodiagnostic testing, repeat spinal imaging, and early rehabilitation may help identify patients who develop Guillain-Barré syndrome-like features and may benefit from immunomodulatory therapy when clinically appropriate. Further studies are needed to better define risk factors for evolving central and peripheral nervous system involvement after WNV infection and to develop evidence-based strategies for monitoring and management.

## Conclusions

This case highlights a rare sequential presentation of neuroinvasive West Nile virus infection, initially manifesting as acute flaccid myelitis and later demonstrating features consistent with Guillain-Barré syndrome-like polyradiculoneuropathy. In medically complex older patients, early recognition of neuroinvasive disease, serial neurologic assessment, and timely rehabilitation are essential to limit long-term functional impairment. Further research is needed to clarify the mechanisms underlying combined central and peripheral nervous system involvement in West Nile virus infection and to guide strategies for prevention, monitoring, and management.
